# Knowledge and Preventive Practices Regarding Nonsteroidal Anti-inflammatory Drug (NSAID)-Induced Kidney Injury Among Chronic Users in Jordan: A Cross-Sectional Study

**DOI:** 10.7759/cureus.108148

**Published:** 2026-05-02

**Authors:** Yahia Atef Albashtawi, Zaina Rawashdeh, Jihan B Jerjeous, Qais Shaban, Lujaien A Alwaked, Hala Hindiyeh, Dana A Al-Jarrah, Manaf Alibraheem, Lubna Al-Raqqad, Mina H Baaji

**Affiliations:** 1 Urology, Abdali Hospital, Amman, JOR; 2 College of Medicine, University of Jordan, Amman, JOR; 3 College of Medicine, The Hashemite University, Zarqa, JOR; 4 College of Medicine, Yarmouk University, Irbid, JOR; 5 College of Medicine, Jordan University of Science and Technology, Irbid, JOR

**Keywords:** chronic use, jordan, kidney injury, misuse, nonsteroidal anti-inflammatory drugs, nsaids, pain

## Abstract

Background

Nonsteroidal anti-inflammatory drugs (NSAIDs) are widely used for acute and chronic pain. However, inappropriate use is a well-established risk factor for kidney injury. Community awareness and preventive practices among chronic users remain unclear. This study aimed to assess knowledge and preventive practices regarding NSAID-induced kidney injury among chronic NSAID users and to examine the association between these two domains.

Methods

A cross-sectional study was conducted (November-December 2025) using an anonymous online questionnaire distributed via social media. Adults (≥18 years) with chronic pain reporting regular NSAID use were eligible. Knowledge (0-6) and preventive practice (0-12) scores were calculated. The association was evaluated using Kendall's tau-b.

Results

Among 300 participants, the mean knowledge score was 3.97 ± 1.24 (95% CI: 3.83-4.11), and the mean practice score was 8.54 ± 2.15 (95% CI: 8.30-8.78). The most deficient knowledge item was awareness of asymptomatic kidney injury (51.3% correct). The weakest preventive practices were reading medication leaflets (54.0% reporting "Always") and avoiding NSAIDs during dehydration (56.7% reporting "Always"). A significant positive association was observed between knowledge and practice scores (τ = 0.317, p < 0.001).

Conclusion

Knowledge is significantly associated with safer NSAID use. Targeted education, particularly regarding asymptomatic injury, hydration, and leaflet reading, may reduce preventable renal harm. Pharmacist-led counseling at the point of sale is a recommended action.

## Introduction

Nonsteroidal anti-inflammatory drugs (NSAIDs) are widely prescribed and frequently purchased over the counter for acute and chronic pain. Their efficacy has led to widespread use globally. However, NSAID-induced kidney injury remains a major cause of preventable renal morbidity worldwide [1,2]. NSAIDs impair renal autoregulation by inhibiting cyclooxygenase-mediated prostaglandin synthesis, reducing renal blood flow, especially during dehydration or underlying vulnerability [3].

The risk increases with prolonged use, high dosing, concurrent nephrotoxic medications, and inadequate fluid intake [4]. Importantly, early injury may be asymptomatic, delaying recognition [5]. Epidemiological studies confirm strong associations with acute kidney injury and chronic kidney disease progression [6]. Despite these risks, public awareness remains limited. Many self-medicate without supervision, particularly in low- and middle-income countries [7]. Preventive behaviors, including hydration, dose limitation, and seeking medical advice, are poorly characterized in community settings [8].

What remains unknown is the level of awareness and specific preventive practices among chronic NSAID users themselves, particularly in Jordan. While clinical outcomes have been studied, community-based behavioral data are sparse. Understanding these gaps is essential to designing effective educational interventions. Therefore, this study aimed to assess knowledge and preventive practices regarding NSAID-induced kidney injury among chronic NSAID users and to examine the association between these two domains.

## Materials and methods

Study design and setting

A cross-sectional observational study was conducted between November 25 and December 28, 2025, using an anonymous, self-administered online questionnaire.

Sample size

A total of 300 participants were included. With a sample of 300, the margin of error is approximately ±5.7% at a 95% confidence level (assuming maximum variability), which was considered acceptable for an exploratory cross-sectional study of this nature.

Participants

Adults (≥18 years) self-reporting chronic NSAID or analgesic use for pain were included. Incomplete responses or non-users were excluded.

Data collection

Participants were recruited using convenience sampling via social media platforms and messaging applications. The response rate could not be calculated precisely due to the anonymous, open-link distribution method, which does not track the number of individuals who viewed the invitation without clicking the link. This is an inherent limitation of the recruitment strategy and has been acknowledged as such.

The questionnaire was developed based on a review of previously published literature assessing NSAID-related knowledge and medication safety practices. It was reviewed by two independent healthcare professionals to ensure content validity and clarity. A pilot test was conducted on a small sample of participants (n = 15) to assess readability and comprehension, and minor modifications were made accordingly.

The questionnaire consisted of sections addressing sociodemographic characteristics, NSAID use patterns (including type, frequency, and duration), knowledge related to NSAID-induced kidney injury, and preventive practices (see Appendices). Knowledge items assessed participant awareness of renal risks associated with NSAID use, dehydration, and prolonged dosing. Preventive practice items evaluated behaviors such as hydration, dose limitation, and consultation with healthcare professionals.

Scoring system

Knowledge scores were calculated by assigning one point for each correct response (Yes/No format), yielding a total score ranging from 0 to 6. Preventive practice items were assessed using a three-point Likert scale (Always = 2, Sometimes = 1, Never = 0), resulting in a total possible score ranging from 0 to 12. Higher scores indicated greater awareness and safer practices.

Ethical considerations

The study involved minimal risk and did not include patient identifiers, clinical interventions, or access to medical records. Electronic informed consent was obtained prior to participation. Ethical approval was not required based on local institutional guidelines for anonymous, minimal-risk survey research. A formal ethics exemption was documented prior to study initiation.

Statistical analysis

Data were analyzed using JASP (Jeffreys’s Amazing Statistics Program; version 0.18.3; University of Amsterdam, Amsterdam, The Netherlands). Descriptive statistics (frequencies, means ± standard deviation, 95% confidence intervals) were calculated. Kendall's tau-b correlation was used to assess the knowledge-practice association. A p-value <0.05 was considered statistically significant.

## Results

Participant characteristics

A total of 300 participants were included. Demographics are shown in Table 1. The sample was predominantly female (52.7%), aged 25-34 years (33.3%), and from Jordan (70.0%).

**Table 1:** Sociodemographic Characteristics of Adult Chronic NSAID Users With Pain (N = 300) NSAID: nonsteroidal anti-inflammatory drug

Variable	Category	N (%)
Age (years)	18-24	69 (23.00)
	25-34	100 (33.33)
	35-44	59 (19.67)
	45-54	48 (16.00)
	≥55	24 (8.00)
Sex	Male	142 (47.33)
	Female	158 (52.67)
Country of residence	Jordan	210 (70.00)
	Other countries	90 (30.00)
Educational level	< High school	21 (7.00)
	High school	86 (28.67)
	Diploma	37 (12.33)
	Bachelor	112 (37.33)
	Postgraduate	44 (14.67)
Employment status	Student	82 (27.33)
	Employed	131 (43.67)
	Unemployed	61 (20.33)
	Retired	26 (8.67)

Patterns of NSAID and analgesic use

Reported analgesic use patterns among participants are demonstrated in Table 2. Paracetamol (62.7%) and ibuprofen (49.3%) were the most common. Over half (56.0%) obtained NSAIDs over-the-counter. Duration of use exceeded six months in 35.0%. Frequency was "as needed" in 31.0%.

**Table 2:** Patterns of NSAID and Analgesic Use Among Adult Participants With Chronic Pain (N = 300) NSAID: nonsteroidal anti-inflammatory drug

Variable	Category	n (%)
Duration of NSAID use	<1 month	42 (14.00)
	1 to <3 months	61 (20.33)
	3 to 6 months	92 (30.67)
	>6 months	105 (35.00)
Frequency of NSAID use	Daily	63 (21.00)
	Several times per week	88 (29.33)
	Weekly	56 (18.67)
	As needed	93 (31.00)
Source of NSAIDs	Over-the-counter	168 (56.0)
	Physician	92 (30.7)
	Pharmacist	40 (13.3)
Dose pattern	Lowest effective dose	182 (60.7)
	Higher than recommended	68 (22.7)
	Not sure	50 (16.7)
Paracetamol use	Yes	188 (62.67)
	No	112 (37.33)
Ibuprofen use	Yes	148 (49.33)
	No	152 (50.67)
Diclofenac use	Yes	119 (39.67)
	No	181 (60.33)
Naproxen use	Yes	74 (24.67)
	No	226 (75.33)
Aspirin use	Yes	70 (23.33)
	No	230 (76.67)

Knowledge and preventive practices

The mean knowledge score was 3.97 ± 1.24 (95% CI: 3.83-4.11), and the mean preventive practice score was 8.54 ± 2.15 (95% CI: 8.30-8.78). The poorest knowledge item was awareness that kidney injury may occur without symptoms (51.3% correct). The weakest preventive practices included reading medication leaflets (54.0% reporting “Always”) and avoiding NSAIDs during dehydration (56.7% reporting “Always”) (Table 3).

**Table 3:** Knowledge and Preventive Practices Related to NSAID-Induced Kidney Injury Among Study Participants (N = 300) NSAID: nonsteroidal anti-inflammatory drug

Domain	Item	Yes/Correct, n (%)	No/Incorrect, n (%)
Knowledge	NSAIDs can harm kidney function	192 (64.00)	108 (36.0)
	Long-term NSAID use increases kidney risk	215 (71.67)	85 (28.33)
	Dehydration increases NSAID-related kidney injury	198 (66.00)	102 (34.00)
	Elderly individuals are at higher risk	187 (62.33)	113 (37.67)
	Diabetes or hypertension increases kidney risk	195 (65.00)	105 (35.00)
	Kidney injury from NSAIDs may occur without symptoms	154 (51.3)	146 (48.7)
Practice	Uses the lowest effective NSAID dose	188 (62.7)	112 (37.3)
	Avoids NSAIDs during dehydration	170 (56.7)	130 (43.3)
	Reads the medication leaflet	162 (54.0)	138 (46.0)
	Consults a healthcare professional	185 (61.7)	115 (38.3)
	Avoids using multiple NSAIDs simultaneously	174 (58.0)	126 (42.0)
	Stops NSAIDs once symptoms improve	192 (64.0)	108 (36.0)

Association between knowledge and practice

**Figure 1:**
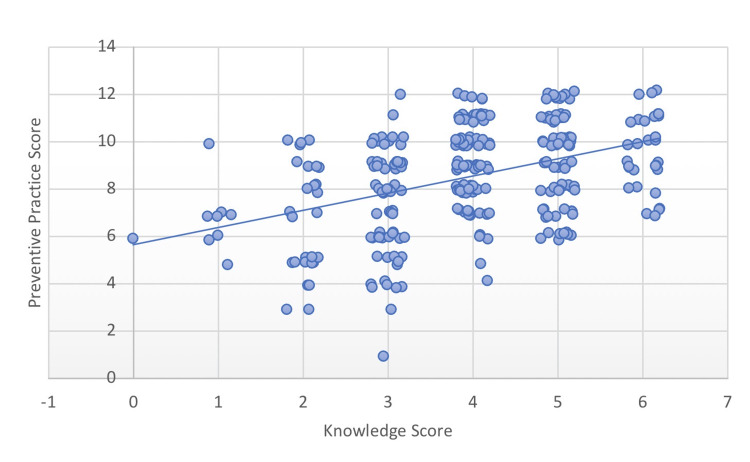
Correlation Between Knowledge and Preventive Practice Scores Related to NSAID-Induced Kidney Injury (N = 300) Scatter plot with jitter demonstrating the association between knowledge and preventive practice scores. A linear regression line is included to aid visualization. Overlapping data points are dispersed using jitter to improve clarity. Kendall's tau-b showed a significant positive correlation (τ = 0.317, p < 0.001). NSAID: nonsteroidal anti-inflammatory drug

The association between knowledge and preventive practice scores is shown in Table 4. A statistically significant positive correlation was observed (Kendall's tau-b = 0.317, p < 0.001), indicating that higher knowledge levels were associated with safer NSAID use behaviors. This relationship is further visualized in Figure 1.

**Table 4:** Correlation Between Knowledge and Preventive Practice Scores (N = 300) Kendall's tau-b correlation showed a statistically significant positive association between knowledge and preventive practice scores (τ = 0.317, p < 0.001).

Variable	Mean ± SD	Minimum	Maximum
Knowledge score	3.97 ± 1.24	0	6
Preventive practice score	8.54 ± 2.15	1	12

## Discussion

This cross-sectional study demonstrated moderate levels of awareness and preventive practices regarding NSAID-induced kidney injury among chronic NSAID users. Notably, higher preventive practice scores were significantly associated with higher knowledge levels, suggesting that better-informed individuals are more likely to engage in safer medication behaviors.

The widespread use of paracetamol and NSAIDs observed in this study is consistent with previous regional and global reports, reflecting their accessibility and frequent use without medical supervision [9,10]. However, awareness of asymptomatic or "silent" kidney injury remains limited, with only 51.3% of participants recognizing that kidney injury may occur without symptoms. This knowledge gap is clinically significant, as patients who do not perceive immediate symptoms may continue unsafe NSAID use, allowing renal damage to progress unrecognized. Similar limitations have been reported in prior community-based studies [11].

The finding that only 54.0% of participants reported always reading medication leaflets represents a concerning gap in preventive practice. This likely reflects several factors common in the Jordanian context: over-the-counter availability reduces perceived need for caution, Arabic-language information on imported NSAID packages is often insufficient, and time pressure during pharmacy purchase discourages leaflet review. Similarly, the low rate of avoiding NSAIDs during dehydration (56.7% reporting "Always") is particularly worrisome given that dehydrated patients are at the highest risk for NSAID-induced acute kidney injury.

The significant association between knowledge and preventive practices (τ = 0.317, p < 0.001) underscores the importance of patient education as a key strategy for reducing NSAID-related harm. Improving patient understanding of renal risks, particularly regarding hydration, appropriate dosing, and duration of use, may yield measurable behavioral changes, consistent with prior medication safety research [12,13]. From a public health perspective, the over-the-counter availability of NSAIDs in Jordan further emphasizes the need for targeted interventions. With 56.0% of participants obtaining NSAIDs without a prescription, pharmacist-led counseling at point-of-sale is essential for reaching this population outside physician oversight.

Several limitations should be considered. The cross-sectional design precludes causal inference. Self-reported data introduce potential recall bias. Online survey distribution may limit generalizability to populations with restricted internet access. Convenience sampling may have introduced selection bias, as more health-aware individuals may have participated. The response rate could not be calculated due to the anonymous distribution method. Despite these limitations, the sample size of 300 enhances the reliability of the findings. Future research should employ longitudinal designs to assess causal relationships and evaluate pharmacist-led educational interventions.

## Conclusions

Chronic NSAID users in Jordan demonstrated moderate awareness and preventive practices regarding NSAID-induced kidney injury, with a significant positive association identified between knowledge and safer medication behaviors. The most critical gaps were a lack of awareness that kidney injury can occur without symptoms, failure to read medication leaflets, and continued NSAID use during dehydration. These findings indicate that improving patient knowledge is a key modifiable factor in promoting appropriate NSAID use and preventing renal harm. Specific actionable recommendations include pharmacist-led counseling at point-of-sale for over-the-counter NSAID purchases, patient education materials highlighting asymptomatic injury risk distributed with every NSAID package, public health campaigns targeting the identified gaps, and enhanced medication leaflets with clear Arabic-language warnings about renal risks and dehydration. Targeted educational initiatives addressing these specific gaps may reduce preventable kidney injury among chronic NSAID users in Jordan and similar settings.

## Appendices

Structured questionnaire used for data collection

**Table 5:** Study Data Collection Instrument

Section	Question	Response Options
A. Eligibility & Consent	Age	18-24/25-34/35-44/45-54/≥55
	Country of residence	Jordan/Qatar/Saudi Arabia/UAE/Kuwait/Other
	City of residence	Amman/Zarqa/Irbid/Balqa/Madaba/Other (Jordan)/Not applicable
	Do you use pain-relief medications for recurring or long-term pain?	Yes/No (If No → End)
	Duration of use	<1 month/1 to <3 months/3-6 months/>6 months
	Consent	I agree to participate
B. Sociodemographic Characteristics	Sex	Male/Female
	Education
	Employment	Employed/Unemployed/Student/Retired
C. Medication Use	Medications used	Paracetamol/Ibuprofen/Diclofenac/Naproxen/Aspirin/Not sure
	Frequency	Daily/Several times/Week/Weekly/As needed
	Source	OTC/Physician/Pharmacist
	Dose	Lowest effective/Higher/Not sure
D. Knowledge	NSAIDs cause kidney injury	Yes/No/Not sure
	Long-term risk	Yes/No/Not sure
	Dehydration risk	Yes/No/Not sure
	Elderly risk	Yes/No/Not sure
	DM/HTN risk	Yes/No/Not sure
	Asymptomatic injury	Yes/No/Not sure
E. Preventive Practices	Use lowest dose	Always/Sometimes/Never
	Avoid NSAIDs when dehydrated	Always/Sometimes/Never
	Read leaflet	Always/Sometimes/Never
	Consult doctor/pharmacist	Always/Sometimes/Never
	Avoid combining NSAIDs	Always/Sometimes/Never
	Stop when improved	Always/Sometimes/Never
F. Source of Information	Main source	Physician/Pharmacist/Internet/Family/None

Consent Statement

You are invited to participate in a research study assessing awareness, knowledge, and preventive practices related to pain-relief medications and kidney health. Participation is completely voluntary. This survey is anonymous, and no personal or identifying information will be collected. There are no anticipated risks associated with participation. You may choose not to answer any question or withdraw from the survey at any time without any consequences. The information collected will be used solely for academic and research purposes.
